# Mesenchymal Conversion of Mesothelial Cells Is a Key Event in the Pathophysiology of the Peritoneum during Peritoneal Dialysis

**DOI:** 10.1155/2014/473134

**Published:** 2014-01-23

**Authors:** Manuel López-Cabrera

**Affiliations:** Centro de Biología Molecular-Severo Ochoa, CSIC, UAM, Cantoblanco, C/Nicolás Cabrera 1, 28049 Madrid, Spain

## Abstract

Peritoneal dialysis (PD) is a therapeutic option for the treatment of end-stage renal disease and is based on the use of the peritoneum as a semipermeable membrane for the exchange of toxic solutes and water. Long-term exposure of the peritoneal membrane to hyperosmotic PD fluids causes inflammation, loss of the mesothelial cells monolayer, fibrosis, vasculopathy, and angiogenesis, which may lead to peritoneal functional decline. Peritonitis may further exacerbate the injury of the peritoneal membrane. In parallel with these peritoneal alterations, mesothelial cells undergo an epithelial to mesenchymal transition (EMT), which has been associated with peritoneal deterioration. Factors contributing to the bioincompatibility of classical PD fluids include the high content of glucose/glucose degradation products (GDPs) and their acidic pH. New generation low-GDPs-neutral pH fluids have improved biocompatibility resulting in better preservation of the peritoneum. However, standard glucose-based fluids are still needed, as biocompatible solutions are expensive for many potential users. An alternative approach to preserve the peritoneal membrane, complementary to the efforts to improve fluid biocompatibility, is the use of pharmacological agents protecting the mesothelium. This paper provides a comprehensive review of recent advances that point to the EMT of mesothelial cells as a potential therapeutic target to preserve membrane function.

## 1. Introduction

Peritoneal dialysis (PD) is a form of renal replacement therapy that has become an established alternative to hemodialysis [1–3]. During the last years, great effort was made to improve the biocompatibility of the dialysis solutions with the expectancy of diminishing their adverse effects on peritoneal morphology and function [4–12]. The number of patients included in PD programs has increased progressively worldwide and is presently used by approximately 10 to 15% of the total global dialysis population [2, 13]. PD offers major advantages in terms of quality of life, costs, and home-based treatment opportunities. The increase of PD programs could also be attributed to the undoubted improvement of the PD technique, especially in terms of peritonitis prevention and of biocompatibility of the dialysis solutions. At present, PD is a successful treatment for end-stage renal disease, and several studies have confirmed equivalent adequacy, mortality, and fluid balance status when compared with hemodialysis, at least for the first 4-5 years [14–17]. However, the growth of PD continues being limited by the membrane incapacity to perform adequate diffusive and/or convective transports at long term [2, 18]. Peritonitis and ultrafiltration failure, with a clinical result of extracellular volume overload and an increased cardiovascular risk, are still the major factors contributing to technique dropouts [2, 18, 19].

PD technique requires the instillation and periodical renovation, through a permanently installed catheter, of a hyperosmotic PD fluid into the peritoneal cavity. The peritoneum acts as a semipermeable membrane across which ultrafiltration and diffusion take place [1–3]. In consequence, one of the most important goals in PD is the long-term preservation of the peritoneal membrane integrity [2, 18, 19]. The use of solutions with neutral pH and with low content of glucose degradation products (GDPs) may represent a potential strategy to attenuate some of the PD-related adverse effects [20]. The impact of these novel, more biocompatible, solutions on the clinical outcomes is currently being recognized [21, 22]. However, classical glucose-based PD fluids are still needed, because the new-generation biocompatible solutions are expensive and many potential users cannot afford them. One possibility to reduce the adverse effects of classical PD fluids on the peritoneum is by decreasing the dwell time of the dialysate [23, 24]. Another alternative approach to preserve the peritoneal membrane could be the use of pharmacological agents protecting the mesothelium or targeting inflammation and fibrosis [25, 26]. In this review, we discuss two putative long-term pharmacological intervention strategies that have been tested in experimental animal models of PD. One strategy is the addition of pharmacological agents into the PD fluids and the other strategy is the use of drugs that are administrated by oral route. We summarize the current knowledge regarding the therapeutic approaches in experimental PD models directed against the epithelial to mesenchymal transition (EMT) of mesothelial cells (MCs) or against the EMT-promoting stimuli operating *in vivo*.

## 2. Pathogenesis of Peritoneal Membrane Dysfunction

The structure of the peritoneum is simple and is composed of a single layer of MCs that lines a compact zone of connective tissue that contains few fibroblasts, mast cells, macrophages, and vessels [27, 28]. It was generally believed that the uremic status might affect the architecture of the peritoneal membrane and its transport characteristics. In this context, the peritoneum of partially nephrectomized rats showed altered permeability [29]. Despite these findings in animal models, the effect of uremia itself on the peritoneum in humans is controversial. Two human peritoneal biopsy studies have shown a modest compact zone thickening and vasculopathy in predialysis renal patients [30, 31]. In contrast, in other studies no significant fibrosis or vasculopathy was observed in uremic non-PD patients [32].

**Figure 1:**
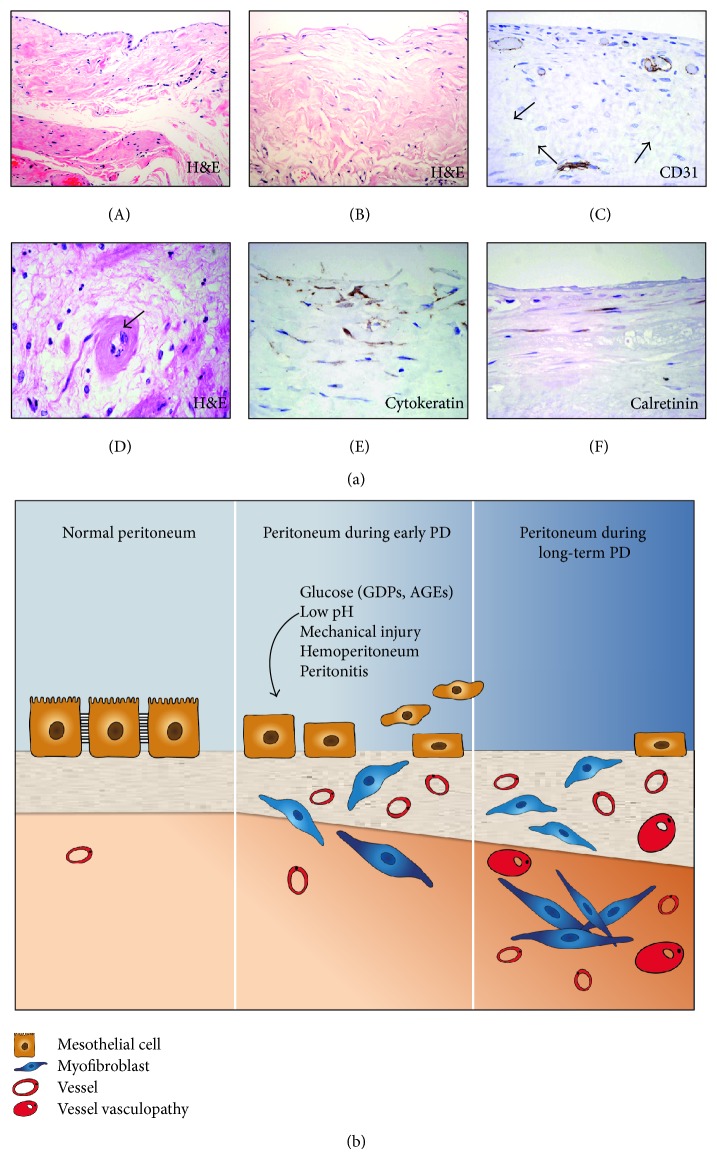
Structural alteration of the peritoneal membrane during PD. (a) Normal peritoneal tissue from a healthy donor stained with Haematoxylin-eosin (H&E) shows a preserved MCs monolayer that lines a compact zone of connective tissue (A). Peritoneal membrane from a PD patient stained with H&E shows the loss of the MCs monolayer and increased thickness of the compact zone (B). Magnification ×200. Staining of the peritoneal vessels with anti-CD31 antibody demonstrates an intense angiogenesis in peritoneal membrane from PD patient (C). Hyalinizing vasculopathy can be observed in the peritoneal tissue from PD patient (D). Immunohistochemical analysis of the peritoneal membrane from PD patient reveals the presence of fibroblast-like cells embedded in the fibrotic stroma expressing the mesothelial markers cytokeratins and calretinin (E) and (F). Magnification ×150. (b) Schematic representation of the progressive alterations of the peritoneal membrane in the time course of PD.

The bioincompatible nature of some PD fluids and episodes of bacterial and fungal infection are considered the main etiologic factors of peritoneal deterioration [2, 12, 19, 25, 33]. They induce acute and chronic inflammatory and reparative responses that initiate the structural alterations of the peritoneal membrane including loss of MCs monolayer, fibrosis, angiogenesis, and hyalinizing vasculopathy [30, 31, 34–36] (Figure 1). Such alterations are considered the major cause of ultrafiltration failure and loss of the dialytic capacity of the peritoneum [2, 19, 37, 38]. There is emerging evidence suggesting that the local injury induced by classical glucose-based PD fluids is mediated, at least in part, by the presence of GDPs and by the acidic pH. GDPs through the formation advanced glycation-end products (AGEs) may stimulate the production of extracellular matrix components (ECM) as well as the synthesis of profibrotic and angiogenic factors [2, 19]. Several studies have demonstrated the appearance of AGEs in the peritoneal effluents of PD patients, which correlated with the time on PD treatment. Biopsy studies have confirmed the accumulation of AGEs in the peritoneal tissues of PD patients. The intensity of AGEs accumulation is associated with fibrosis and ultrafiltration dysfunction [2, 19].

The peritoneal immune response to injury or infection involves, among other cells, MCs and resident macrophages that work in a coordinated manner to recruit other inflammatory cells, including mononuclear phagocytes, lymphocytes, and neutrophils. MCs and infiltrating immune cells can produce a wide number of cytokines, growth factors, and chemokines to establish a complex network that feedbacks resulting in acute or chronic inflammation [2, 25, 39–41]. Sustained inflammation might trigger the fibrogenic and angiogenic processes associated with the ultrafiltration failure that causes PD technique dropout (Figure 1).

There are two different pathologic forms of PD-related fibrosis [42–44]. The most common is simple peritoneal sclerosis (SPS), which occurs in almost all patients. The degree of fibrosis is mild and shows a relation with the time on dialysis. In general terms, SPS ceases when the patient is transplanted or shifted to hemodialysis [28, 31, 38, 42–44]. On the other end of the spectrum is encapsulating peritoneal sclerosis (EPS), which is a rare form of sclerosis that evolves rapidly with intense fibrosis, inflammation, and fibrin deposits [43–46]. It is a life threatening condition that in many cases evolves to visceral encapsulation with a fibrous cocoon and progresses even if the patient is removed from PD. In this context, EPS often becomes apparent after renal transplantation or switching patients to hemodialysis [47–49]. The etiopathogenesis of EPS is still debated, with some sustaining that it is a rare form of progression of SPS and others that it is a primitive form of sclerosis [50–52]. Thus, the main reasons that have led to PD-induced sclerosis to become a subject of active research are the high frequency of mild degree peritoneal fibrosis (SPS) and the severity and poor prognosis of EPS.

However, fibrosis does not appear to be the unique structural alteration of the peritoneal membrane induced by PD. Besides this alteration the peritoneum may also show an increase of capillary number (angiogenesis) and hyalinizing vasculopathy [2, 25] (Figure 1). Vascular endothelial growth factor (VEGF) is a strong angiogenic factor involved, among other molecules, in endothelial cell proliferation and vascular permeability [53]. It has been suggested that local production of VEGF during PD plays a central role in the processes leading to peritoneal angiogenesis and functional decline [54–58]. It has been demonstrated that MCs can produce high amounts of VEGF *in vitro* in response to various stimuli [59–62]. In addition, it has been suggested that MCs, via a mesenchymal conversion, may convert into the major local producer of VEGF during PD, which in turn appears to be associated with peritoneal transport alteration [57, 62, 63]. Some studies of peritoneal biopsies have suggested that angiogenesis and vasculopathy are the most characteristic structural alteration in PD-related peritoneal pathology, at least in patients with severe membrane failure [30, 34]. In contrast, other studies have shown that in stable uncomplicated PD patients vascular density does not increase, while intact vessels decrease with time of treatment and severe vasculopathy predominate mostly in long-term PD [36, 64, 65].   The only change that is constantly found in peritoneal biopsies after a time on PD is submesothelial fibrosis [2, 19, 30–32, 34]. Nevertheless, there is increasing evidence that fibrosis in conjunction with angiogenesis and most probably with augmented vessel permeability are key determinants of ultrafiltration dysfunction [25, 30]. In animal models of PD it has been shown that fibrosis and angiogenesis may be two separate responses to peritoneal injury [66–69]. However, in PD patients, it is possible that fibrosis and angiogenesis are intimately and closely related in the response of the peritoneum to prolonged injury [25].

## 3. Peritoneal Dialysis Induces the Accumulation of Myofibroblasts

Another characteristic structural alteration of the peritoneum during PD is the loss of the MC monolayer and the progressive accumulation of a particular type of activated fibroblast termed myofibroblast (Figure 1), which, as will be discussed below, derive partially from the local conversion of MCs. The term myofibroblast defines a cell with intermediate features between a fibroblast and a smooth muscle cell. From an immunophenotypic perspective, they are defined by the expression of *α*-smooth muscle actin (*α*-SMA). Myofibroblasts were initially described by Gabbiani et al. in the granulation tissue of a cutaneous model of wound repair [70, 71]. Since then, they have been reported as important protagonists of almost all situations of repair and fibrosis that take place in human pathology [72]. Their capacity to synthesize extracellular matrix elements, growth factors, cytokines, and participation in the inflammatory response, as well as their contractile properties, converts them to the most important fibroblastic phenotype. As stated by Phan they must be considered the “reference” fibroblast phenotype to which all others must be related or compared [73]. Myofibroblasts are neither present in the normal peritoneum nor in the peritoneum obtained from uremic non-PD patients [32, 74]. In contrast, they can be easily observed in many patients undergoing PD treatment [32, 34, 75].

**Figure 2:**
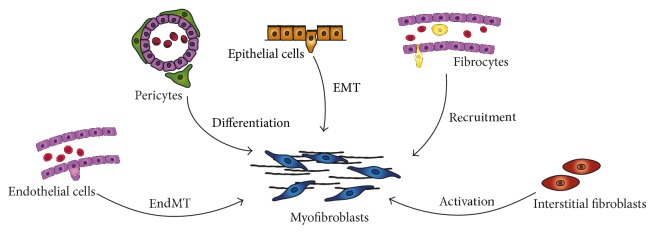
Multiple origins of myofibroblasts have been proposed in tissue fibrosis. Myofibroblasts may derive from at least five different sources through various mechanisms: phenotypic activation from interstitial fibroblasts; differentiation from vascular pericytes; recruitment from circulating fibrocytes; capillary endothelial-mesenchymal transition (EndMT); and epithelial-mesenchymal transition (EMT). The relative contribution of each source to the myofibroblast pool in peritoneal fibrosis still requires further studies.

The origin of myofibroblasts is still an open question and a matter of intense debate [76–81], but it is generally accepted that these fibroblasts constitute a heterogeneous population that may derive from multiple sources (Figure 2). There is emerging evidence that the origin of myofibroblasts may vary between different organs and within different areas of individual organs. These observations may suggest that tissue- and organ-specific microenvironments dictate the different proportions of myofibroblasts subpopulations [76, 77, 82–87]. The activation of resident fibroblasts has classically been considered the main origin of myofibroblasts in most fibrotic pathologies [70–73, 83, 86]. Other studies have pointed to cells recruited from the bone marrow (fibrocytes) as an important source of myofibroblasts in several fibrotic disorders [76, 87–90]. In addition, it has been shown that the local conversion of epithelial cells and endothelial cells may also contribute to the accumulation of myofibroblasts in some reparative and fibrotic diseases. The conversion into myofibroblasts by these cells is achieved through two closely related processes termed epithelial to mesenchymal transition (EMT) and endothelial to mesenchymal transition (EndMT), respectively [86, 90–95]. More recently it has been suggested that vessel-associated pericytes may also transdifferentiate into myofibroblasts [77, 96] (Figure 2).

In the peritoneal membrane, the myofibroblasts may have at least a dual origin: (1) from resident fibroblasts through an activation process and (2) from the mesothelium via EMT [25, 32, 97] (Figure 1). The presence of other myofibroblasts subpopulations in the damaged peritoneum during PD has not been described so far in PD patients [25]. However, in a mouse model of PD fluid exposure, it has been shown that myofibroblasts may have different origins including resident fibroblasts, MCs, endothelial cells, and bone marrow-derived cells [82]. As we will discuss below, the identification of the EMT of MCs as a key process in the onset and progression of peritoneal fibrosis and angiogenesis opens new insights for therapeutic intervention.

## 4. Mesothelial Cells Undergo a Mesenchymal Transition in Response to PD-Induced Damage

The mesothelium is a continuous surface layer formed by flattened, polygonal, and mononuclear MCs [28]. This monolayer shows remarkable fibrinolytic properties and is thought to be involved in the prevention of fibrous adhesion formation in the peritoneum. MCs cells have vast biosynthetic capacity and secrete phospholipids and phosphatidylcholine in the form of lamellar bodies that provide a lubricating surface for the movement of abdominal viscera [98–100]. The presence of MCs that have undergone a mesenchymal conversion *in vivo* in the effluent and in the peritoneal tissue of PD patients was first demonstrated in a landmark paper published in 2003 [97]. The authors described that soon after PD is initiated, peritoneal MCs showed a progressive loss of epithelial phenotype and acquired myofibroblast characteristics [97]. About the same time it was demonstrated that the treatment *in vitro* of omentum-derived MCs with TGF-*β*1 induced a myofibroblast conversion of these cells that were reminiscent of an EMT-like process [101].

Effluent-derived MCs can be easily isolated from PD patients using standard methods [97, 102]. It was described that *ex vivo* cultures of effluent-derived MCs showed two main morphologies: epithelioid and nonepithelioid (fibroblast-like). After analyzing several hundred MC cultures with growth capacity, it could be determined that the frequencies of the different effluent-derived MC cultures were approximately 53 percent for epithelioid phenotype and 44 percent for nonepithelioid MCs. The prevalence of nonepithelioid MC cultures appeared to be associated with the time the patients have been subjected to PD and with the episodes of acute or recurrent peritonitis or hemoperitoneum [97, 102]. A less frequent cell culture type (less than 6 percent) with mixed morphologies has also been described [97, 102]. In the course of practicing *ex vivo* cultures of effluent-derived cells, it can be observed occasionally hypertrophic MCs, which might appear alone or accompanied by MCs with a normal size [102, 103]. Hypertrophic MCs could be the consequence of an arrest of the cell cycle, since these cells are unable to proliferate [104].

Proliferating MCs from effluents showed high expression of ICAM-1 independently of their morphology, and even mixed cultures were homogeneous in the expression of this marker. On the contrary, ICAM-1 was negligible on fibroblasts from omentum, supporting that effluent non-epitheliod cells have a mesothelial origin [97]. In addition, effluent-derived cells also showed high expression of CA-125, a known mesothelial marker, independently of their shape, whereas fibroblasts were negative for this molecule, reinforcing the concept of a mesothelial origin of these cells and rule out possible fibroblast contaminations [102]. The analysis of the expression of the epithelial markers cytokeratins and E-cadherin was important to determine more precisely the nature of effluent-derived cells. High expression of cytokeratins and E-cadherin was only observed in naïve omentum-derived MC, whereas effluent-derived cells showed a progressive reduction in the expression of these molecules, although even nonepithelioid MCs might maintain a small population of positive cells. Fibroblasts were completely negative for these two markers [97, 102]. The morphological changes and downregulation of cytokeratin and E-cadherin in effluent-derived MCs were indicative of an EMT-like process. However, the definitive prove to demonstrate that the PD-induced phenotype changes of the MCs were related with an EMT process came from the analysis of the expression of several mesenchymal markers including snail, N-cadherin, fibronectin, collagen I, *α*-smooth-muscle actin (*α*-SMA), and fibroblast specific protein-1 (FSP-1) that were gradually upregulated in effluent MCs with epithelioid and nonepithelioid phenotypes [19, 25, 97, 102].

MCs that have undergone a mesenchymal phenotype acquire higher migratory and invasive capacities, which allow these cells to invade the submesothelial stroma [25, 58, 97, 105]. Thus, the mesenchymal conversion of MCs may also be observed *in vivo* in the peritoneum as a response to PD. Immunohistochemical analysis of peritoneal biopsies from PD patients has shown the presence of fibroblast-like cells embedded in the compact zone expressing mesothelial markers such as cytokeratins, E-cadherin, ICAM-1, and calretinin [25, 32, 57, 65, 97] (Figure 1(a)). In addition, these peritoneal biopsies showed expression of *α*-SMA in the fibrotic stroma, especially in the upper submesothelial level, and in many cases these myofibroblasts showed coexpression of cytokeratins [32, 74]. These results indicated that new myofibroblastic cells could arise from local conversion of MCs by EMT during the repair responses that take place in PD. The myofibroblastic conversion of MC has been confirmed in an *in vivo* animal model based on the injection of an adenovirus vector that transferred active transforming growth factor (TGF)-*β*1 in rodent peritoneum [106, 107].

**Figure 3:**
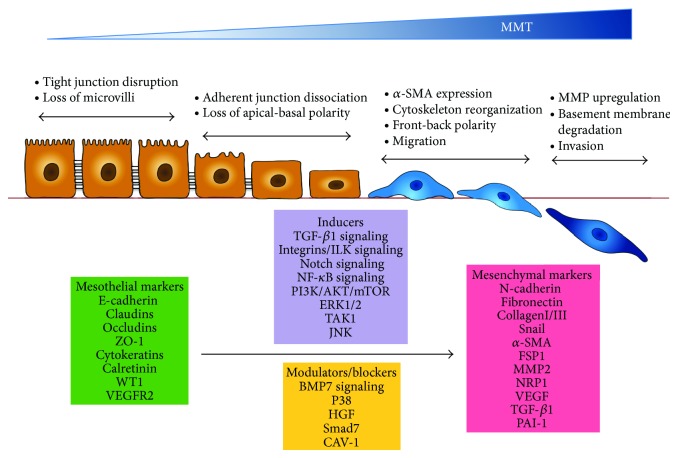
Schematic illustration of the key events during MMT. Mesothelial to mesenchymal transition (MMT) occurs when mesothelial cells lose their epithelial-like characteristics, including dissolution of cell-cell junctions, that is, tight junctions, adherens junctions and desmosomes, and loss of apical-basolateral polarity, and acquire a mesenchymal phenotype, characterized by actin reorganization and stress fiber formation, migration, and invasion. The diagram shows four key steps essential for the completion of entire MMT, the most commonly used mesothelial and mesenchymal markers, and the molecules and signal transduction pathways that act either as inducer or modulator of the MMT process. See text for details.

MCs have a mesodermal origin and share characteristics with both epithelial cells and endothelial cells, which may undergo EMT and endothelial to mesenchymal transition (EndMT), respectively. Thus, recently several authors have proposed renaming the mesenchymal conversion of MCs, that takes place in different organs such as lung, liver, or peritoneum, with a more appropriate term: mesothelial to mesenchymal transition (MMT) [62, 82, 108–111]. MMT is a complex and step-wise process that requires alterations in cellular architecture and a deep molecular reprogramming with new biochemical instruction [19, 25, 58]. MMT starts with the dissociation of intercellular junctions, due to downregulation of intercellular adhesion molecules, and with the loss of microvilli and apical-basal polarity. Then, the cells adopt a front to back polarity and acquire *α*-SMA expression and increased migratory capacity. In the latest stages of MMT, the cells increase their capacity to degrade the basement membrane and to invade the fibrotic compact zone (Figure 3). During the end-stages of the myofibroblast conversion, the MCs are able to produce large amount of extracellular matrix components and to synthesize a wide range of inflammatory, profibrotic, and angiogenic factors that may contribute to the structural and functional deterioration of the peritoneal membrane [2, 19, 25, 58]. Other commonly used molecular markers for MMT include the downregulation of cytokeratins, Wilm's tumor protein-1 (WT1), and calretinin and up-regulation of N-cadherin, FSP-1, and transcription factor snail (Figure 3).

MMT process *in vivo* results from an integration of diverse signals triggered by multiple factors, being difficult to assign priorities or hierarchy [25, 58, 63]. Receptors-mediated signaling in response to these factors trigger the activation of a complex network of intracellular effectors such as Smad 2 and 3, integrin-linked kinase (ILK), Notch1, nuclear factor-*κ*B (NF-*κ*B), extracellular-signal regulated kinases 1/2 (ERKs1/2), phosphatidylinositol 3-kinase (PI3-K)/Akt pathway, c-jun-N terminal kinase (JNK), and TGF-*β*-activated kinase-1 (TAK-1) (Figure 3). These effectors orchestrate the dissociation of intercellular adhesion complexes, the changes in cytoskeletal organization, and the acquisition of migratory and invasive capacities that take place during MMT [25, 63, 105, 106, 112, 113].

It is noteworthy that MMT is a reversible process, at least during the early stages. Therefore, molecules that negatively regulate MMT and promote mesenchymal to mesothelial transition (rMMT) must exist. Two endogenous factors, namely, hepatocyte growth factor (HGF) and bone morphogenetic protein-7 (BMP-7), have been demonstrated to block and reverse MMT [114–116]. Smad7 is another molecule that negatively regulates MMT [117–119]. On the other hand, the MMT process may be modulated by mitogen-activated protein (MAP) kinase p38 to prevent an exacerbated response to MMT-promoting stimuli [120]. Recently, it has been shown that caveolin-1 (CAV-1) impedes the exacerbation of the mesenchymal conversion of endothelial cells [121] and MCs (unpublished data) by promoting the internalization of TGF-*β* receptor and modulating TGF-*β* signaling (Figure 3).

## 5. TGF-***β***1 Is a Master Molecule in the Pathogenesis of Peritoneal Damage and in the Regulation of MMT

In the complex microenvironment that occurs during PD fluid-induced tissue injury a wide range of cytokines and factors are upregulated making it difficult to assign priorities or hierarchy for their effects on MMT and on the onset and progression of peritoneal damage [25]. Nonetheless, TGF-*β*1 is considered a master molecule in the development of peritoneal dysfunction, because its overexpression has been correlated with worse PD outcomes [122–124]. The relevance of TGF-*β*1 in peritoneal damage is further suggested in experimental animal models, in which TGF-*β*1 gene is overexpressed into the peritoneal cavity with adenovirus vectors, recapitulating the structural and functional alterations observed in PD patients [106, 107, 125]. Overexpression of molecules counteracting TGF-*β*1-triggered Smad signaling, including Smad7 and BMP-7, prevents and reverses PD fluid induced peritoneal damage in animal PD models [115, 116, 118, 119]. Recently, it has been demonstrated that direct targeting of TGF-*β*1, by using specific TGF-*β*1-blocking peptides, preserves the peritoneal membrane from dialysis fluid-induced damage in a mouse PD model [82]. TGF-*β*1 is a prototypical inducer of EMT in several tissues and organs [126–128]. TGF-*β*1 is also a key factor for the myofibroblastic conversion of MCs through MMT [58, 82, 97, 101].

### 5.1. Smad-Dependent Signaling Pathways in TGF-*β*1-Induced MMT

**Figure 4:**
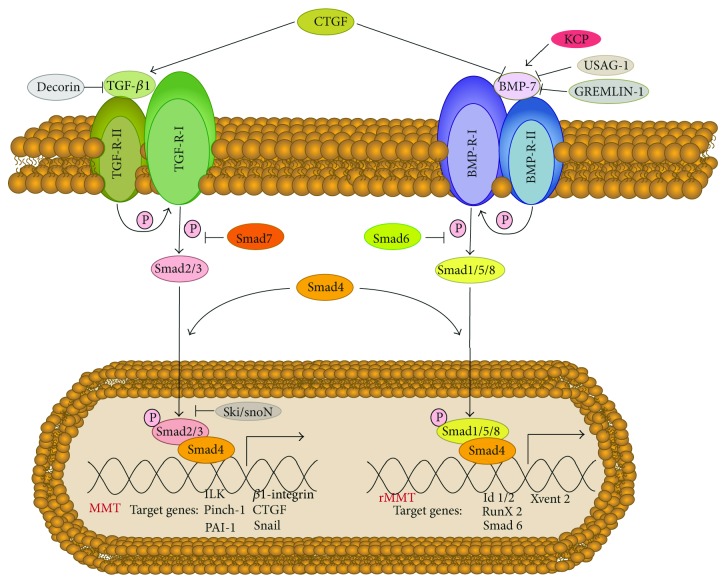
Smad-dependent signaling pathways of TGF-*β*1 and BMP-7. The binding of TGF-*β*1 and BMP-7 to their primary receptors (receptors type II) allows the recruitment, transphosphorylation, and activation of the signaling receptors (receptors type I). The receptor type I of TGF-*β*1 phosphorylate Smad2 and Smad3. The receptor type I of BMP-7 phosphorylate Smad1, Smad5, and Smad8. These receptor-activated Smads form heterodimers with Smad4. The resulting Smad complexes are then translocated into the nucleus where they activate target genes involved either in the mesenchymal conversion of MCs (MMT) in the case of Smads2/3 or in the blocking/reversion of the mesenchymal transition (rMMT) in the case of Smads1/5/8. Smad6 and Smad7 control BMP-7- and TGF-*β*1-triggered Smad signaling by preventing the phosphorylation and/or nuclear translocation and by inducing receptor complex degradation through the recruitment of ubiquitin ligases. Extracellular regulation of TGF-*β*1 and BMP-7 is achieved by various molecules. CTGF inhibits BMP-7 and activates TGF-*β*1 signals by direct binding in the extracellular space. BMP-7 signaling might also be influenced by other BMP-7 modulators such as gremlin-1, kielin/chordin-like protein (KCP), or uterine sensitization-associated gene 1 (USAG-1).

TGF-*β*1 belongs to a family of growth factors that includes TGF-*β*s, activins, and bone morphogenic proteins (BMPs) [126–130]. We will focus on the members TGF-*β*1 and BMP-7 because the balance between these two factors is a key determinant in the maintaining of the epithelial-like phenotype of MCs, and conversely, in the acquisition of mesenchymal-like characteristics [114, 116]. In fact, BMP-7 is a natural antagonist of TGF-*β*1 during organ fibrosis [130]. These factors signal via heterodimeric serine/threonine kinase transmembrane receptor complexes [129–131]. The binding of the ligand to its primary receptor (receptor type II) allows the recruitment, transphosphorylation, and activation of the signaling receptor (receptor type I) (Figure 4). The receptor type I of TGF-*β*1, also known as activin receptor-like kinase 5 (ALK5), is then able to exert its serine-threonine kinase activity to phosphorylate Smad2 and Smad3. The receptor type I of BMP-7 (ALK3) phosphorylate Smad1, Smad5, and Smad8 (Figure 4). These receptor-activated Smads (R-Smads) interact directly with and are phosphorylated by activated TGF-*β* or BMP receptor type I, respectively. Upon phosphorylation, they form heterodimers with Smad4, a common mediator of all Smad pathways [126, 129–131]. The resulting Smad heterocomplexes are then translocated into the nucleus where they bind directly to DNA and activate target genes involved either in the mesenchymal conversion of MCs (MMT) in the case of Smads2/3 or in the blocking/reversion of the mesenchymal transition (rMMT) in the case of Smads1/5/8 (Figure 4). Members of the third group of Smads, known as inhibitory Smads (Smad6 and Smad7), control BMP-7- and TGF-*β*1-triggered Smad signaling by preventing the phosphorylation and/or nuclear translocation of R-Smads and by inducing receptor complex degradation through the recruitment of ubiquitin ligases [126, 127, 129, 130].

The necessity of Smad2/3 signaling in TGF-*β*1-induced MMT is clearly illustrated *in vivo* in Smad3 knockout mice, which are protected from peritoneal fibrosis, show reduced collagen accumulation, and display attenuated MMT [106]. Targeting Smad signaling by inhibitory Smad7 also blocks MMT and reduces peritoneal fibrotic lesions [129–131]. Blockade of Smad2/3 signaling is also linked to the inhibition of MMT by hepatocyte growth factor (HGF) and BMP-7 [115, 116]. Mechanistically, HGF interferes with TGF-*β*1-mediated MMT by inducing the expression of the transcriptional corepressors such as SnoN and TGIF that interact with activated Smad2/4 complex and block the expression of Smad-dependent genes [132–134]. The mechanism underlying BMP-7 blockade of MMT is by activation of Smad1/5/8 protein that counteracts with TGF-*β*-activated Smad2/3 [116, 130].

It has been shown that MCs constitutively express BMP-7 and display basal activation of Smad1/5/8, which probably contribute to the maintaining of the epithelial-like phenotype. Induction of MMT with TGF-*β*1 results in downregulation of BMP-7 and inactivation of BMP-7-specific Smad proteins [116]. Mechanistically, the TGF-*β*1-mediated inhibition of BMP-7 signaling might be explained by BMP-7 downregulation itself, or alternatively, by the upregulation of modulators of BMP-7 and TGF-*β*1 pathways. In this context, it has been shown that connective tissue growth factor (CTGF), a cytokine that is induced in MCs upon TGF-*β*1 treatment [135–138], inhibits BMP-7 and activates TGF-*β*1 signals by direct binding in the extracellular space [139, 140]. In addition, mesothelial BMP-7 signaling might also be influenced by other BMP-7 modulators such as gremlin-1, kielin/chordin-like protein (KCP), or uterine sensitization-associated gene 1 (USAG-1) [130, 141] (Figure 4). Thus, the relative contribution of these different factors in the inhibition of BMP-7 pathway by TGF-*β*1 remains to be established and deserves further studies.

### 5.2. Non-Smad Signaling Pathways in TGF-*β*1-Induced MMT

**Figure 5:**
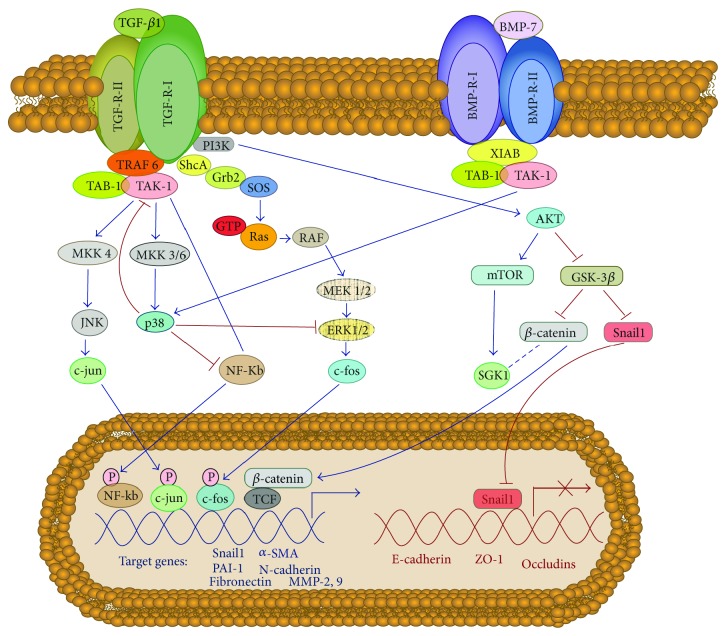
Non-Smad signaling in response to TGF-*β*1 and BMP-7. TGF-*β*1 activates MAP kinases JNK and p38 signaling and NF-*κ*B through the activation of TAK1 by receptor-associated TRAF6. TGF-*β*1 also activates MAP kinase ERK 1/2 signaling through recruitment and phosphorylation of Shc by the TGF-*β*1 type I receptor. In MCs the p38-mediated pathway acts as modulator of the mesenchymal conversion by a feedback mechanism based on the downregulation of ERKs 1/2, NF-*κ*B, and TAK-1 activities. Interestingly, BMP-7 activates p38 signaling by receptor-associated XIAB, which may contribute to the maintaining epithelial-like phenotype. TGF-*β*1 also induces PI3-K/Akt pathway leading to the activation of mTOR and the stabilization of *β*-catenin and snail through the inactivation of GSK-3*β*. As a result, *β*-catenin localizes to the nucleus, where it feeds into the Wnt signaling pathway by interacting with lymphoid enhancer factor-1/T-cell factor (LEF1/TCF) and contributes to the transcription of mesenchymal-related genes. In addition, the nuclear translocation of snail promotes the transcriptional repression of E-cadherin and other intercellular adhesion molecules.

The Smad-dependent pathways are not the only ways by which TGF-*β*1 regulate cellular functions in MCs including the MMT process. Smad-independent pathways including the mitogen-activated protein kinases (MAPKs) ERKs 1/2, JNK, and p38, as well as NF-*κ*B, TAK-1, and PI3-K/Akt pathways, also participate in TGF-*β*1-induced MMT (Figure 5). These pathways can either potentiate or modulate the outcome of TGF-*β*1-induced Smad signaling. Emerging evidences suggest that Smad signaling is tightly integrated within a complex network of signaling pathways with cross-talks that modify the initial Smad signals and allow the pleiotropic activities of TGF-*β*1 [142, 143]. In this context, it has been shown that the signaling pathways of ERKs 1/2, JNK, NF-*κ*B, and TAK-1 potentiate the TGF-*β*1-induced MMT [105, 112]. On the contrary, the p38-mediated pathway modulates the mesenchymal conversion of MCs by a feedback mechanism based on the downregulation of ERKs 1/2, NF-*κ*B, and TAK-1 activities [120] (Figure 5).

There are instances in which Smad signaling is not required for TGF-*β*1 responses, as exemplified by the activation of the PI3-K/Akt pathway in Smad3 deficient mice leading to the stabilization of *β*-catenin, which in turn promote MMT [106]. A central role in this Smad3-independent signaling pathway is achieved by glycogen-synthase kinase (GSK)-3*β*, which has been shown to phosphorylate *β*-catenin and the transcriptional repressor Snail, leading to their ubiquitinization and degradation via the proteasome. The phosphorylation of GSK-3*β* by PI3-K/Akt leads to its functional inhibition. As a result, *β*-catenin is stabilized and localizes to the nucleus, where it feeds into the Wnt signaling pathway by interacting with lymphoid enhancer factor-1/T-cell factor (LEF1/TCF) and contributes to the transcription of mesenchymal-related genes. In addition, the inhibition of GSK-3*β* also drives the stabilization and nuclear translocation of Snail, a potent transcriptional repressor of E-cadherin and other intercellular adhesion molecules [144–147] (Figure 5). Interestingly, *in vivo* inhibition of the mammalian target of rapamycin (mTOR) by rapamycin completely abrogates the MMT response in Smad3-deficient mice [106]. Thus, TGF-*β*1 causes peritoneal injury through Smad-dependent and Smad-independent pathways suggesting that suppression of both pathways may be necessary to abrogate MMT.

## 6. Pathologic Significance of MMT in Peritoneal Dysfunction

It has been shown that during the progression of MMT, MCs acquire the ability to synthesize large amounts of components of the matrix such as fibronectin and collagen I [25, 57, 58]. In addition, MCs that undergo a MMT express high levels of cyclooxygenase (COX)-2 [148, 149], CTGF [135, 136, 138], and VEGF [54, 56, 57], which have been implicated in inflammatory responses as well as in the fibrotic and angiogenic processes [2, 19, 25, 58]. In this context, it has been described that MCs from effluents with non-epitheliod (fibroblast-like) phenotype produced higher levels of COX-2 and VEGF *ex vivo* than MCs with epithelial-like phenotype. Interestingly, the levels of expression of these molecules by cultured effluent MCs correlated with the rate of peritoneal transport in PD patients [57, 148]. In addition, it was observed that patients draining non-epitheliod cells had higher blood VEGF levels than patients with MCs with epithelial-like phenotype in their effluents. Again, a correlation between *in vivo* VEGF levels and the rate of peritoneal transport in PD patients could be demonstrated [57]. A clinical study using peritoneal biopsies from 35 stable patients being on PD for up to 2 years demonstrated that patients in the highest quartile of mass transfer area coefficient of creatinine (Cr-MTAC) showed significantly higher MMT prevalence in the peritoneal compact zone. In the multivariate analysis, the highest quartile of Cr-MTAC remained as an independent factor predicting the presence of MMT after adjusting for fibrosis [65]. These findings indicate that MMT is a frequent morphological change in the peritoneal membrane.

Another study showed that the dialysate-to-plasma ratio for creatinine (D/P Cr) was positively correlated to dialysate CTGF concentration. Furthermore, CTGF mRNA expression was higher in peritoneal tissues with ultrafiltration failure and was correlated with thickness of the peritoneum. Interestingly, the study demonstrated that high peritoneal transport state was associated with increased CTGF production by effluent MCs stimulated with TGF-*β*1 [138]. Thus, these results suggest that functional alteration of MCs, namely, acquisition of mesenchymal properties, may be involved in the progression of peritoneal structural alteration and in high transport state.

## 7. MMT as a Potential Therapeutic Target

**Figure 6:**
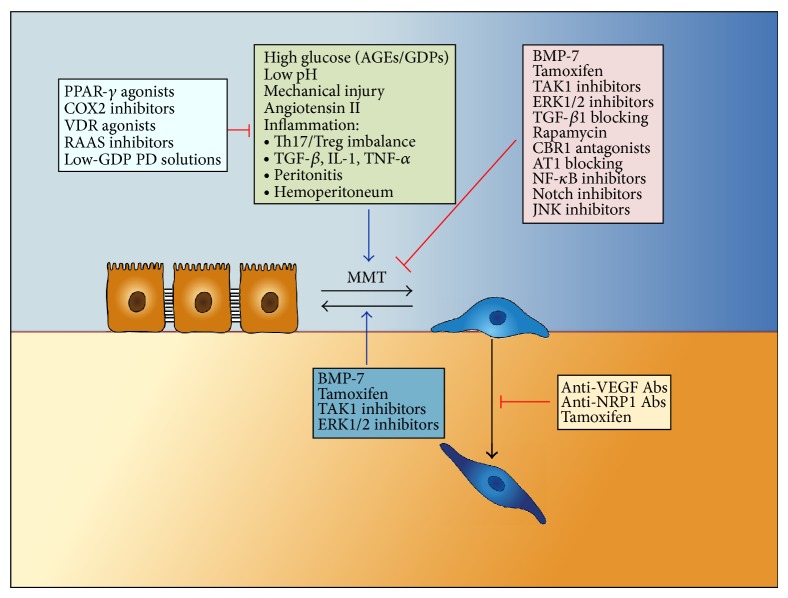
Therapeutic strategies for peritoneal membrane failure based on MMT. MMT *in vivo* results from integrated signals induced by multiple stimuli. These include high concentration of glucose and glucose degradation products (GDPs) in the PD fluids, which contribute to the formation of advanced glycation-end products (AGEs) and stimulate the mesenchymal conversion of MCs. The low pH of the dialysates and the mechanical injury during PD fluid exchanges may cause tissue irritation and contribute to chronic inflammation of the peritoneum, which promote MMT. Episodes of bacterial or fungal infections or hemoperitoneum cause acute inflammation and upregulation of cytokines and growth factors such as TGF-*β*, IL-1, TNF-*α*, and Angiotensin II, among others, which are strong inducers of MMT. The therapeutic strategies may be designed either to prevent or reverse the MMT itself, to decrease the MMT-promoting stimuli, or to treat MMT-associated effects such as the invasion capacity to avoid their accumulation in the compact zone. The diagram illustrates aspects related with the MMT process that can be clinically managed, alone or in combination, in order to prevent peritoneal membrane failure. See text for details.

Having accepted that MMT is a key event in peritoneal damage induced by PD, during the last years it has been suggested that MMT might be a potential target for therapeutic intervention [25, 58]. The therapeutic strategies may be designed to block or revert the MMT itself because this process can be manipulated with a wide range of agents and pharmaceutical products. Conversely, the therapeutic approaches may be directed to interfere or modify the upstream MMT-promoting stimuli operating *in vivo* (e.g., inflammation, low pH, mechanical injury, GDPs content of PD fluids, and accumulation of AGEs) (Figure 6). For the design of the different therapeutic approaches, *in vitro* and *ex vivo* cultures of MCs as well as experimental animal models of PD have been very useful for testing pharmacological agents with potential effects on MMT.

The use of solutions with neutral pH and low GDPs content may represent the first and most obvious approach to attenuate some of the PD-related adverse effects including the mesenchymal conversion of MCs [20–22]. It has been shown that these new-generation low-GDPs fluids have less impact on MMT *in vivo* in PD patients and *in vitro* in cultured MCs [150, 151]. In agreement with these results, it has been demonstrated that low-GDPs fluids induce less inflammatory response and less fibrosis in a mouse PD model [148]. However, completely biocompatible PD fluids will be difficult to develop, at least under cost-effective perspectives. In addition, classical glucose-based PD fluids are still needed, because the new-generation biocompatible solutions are expensive and many potential users cannot afford them. An alternative approach to preserve the peritoneal membrane could be the use of pharmacological agents targeting inflammation and injury or preserving the mesothelium (Figure 6). Two long-term pharmacological intervention strategies have been tested in experimental animal models of PD. One strategy is the addition of pharmacological agents into the PD fluids and the other strategy is the use of drugs that are administrated by oral route.

As discussed above, TGF-*β*1 is a master molecule in the pathogenesis of peritoneal damage and in the regulation of MMT. In fact it has been demonstrated that addition to the PD fluid of two specific TGF-*β*1-blocking peptides preserved the peritoneal membrane from damage in a mouse PD model [82]. However, it should be considered that agents directly blocking TGF-*β*1 cannot be easily employed in the clinical practice of PD, at least for long-term treatments, because TGF-*β*1 has important modulating functions of the immune and inflammatory responses [152, 153]. The molecular studies of the TGF-*β*1 Smad-dependent and Smad-independent signaling pathways involved in MMT provide more specific strategies for the preservation of peritoneal membrane with less side effects (Figure 6). In this context, the endogenous factors HGF and BMP-7 have been demonstrated to block MMT *in vitro*. In addition, intraperitoneal administration of these proteins prevented and reverted peritoneal damage in experimental animal models [115, 116, 154]. It is important to note that the use of BMP-7 may be difficult to be used in the clinical practice of PD because it has been associated with ossification; indeed, BMP-7 has been administered locally into bone lesions to promote bone formation [155]. An alternative to BMP-7 would be the use of synthetic agonists of the BMP-7 receptor ALK3 [156].

Three examples of therapeutic drugs that have demonstrated to preserve the peritoneal membrane after administration by oral route are Celecoxib, Rosiglitazone, and Tamoxifen [108, 111, 148, 149] (Figure 6). Celecoxib is a potent anti-inflammatory drug whose mechanism of action is based on the inhibition of COX-2. In mouse or rat PD models, orally administered Celecoxib decreased peritoneal inflammation, angiogenesis, and fibrosis and preserved peritoneal membrane function [148, 149]. Rosiglitazone is an agonist of the peroxisome proliferator-activated receptor (PPAR)-*γ* that improves insulin sensitivity. The high concentration of glucose and GDPs in standard PD fluids induce a local diabetic environment, which leads to the formation of AGEs that have an important role in peritoneal membrane inflammation. PPAR-*γ* agonists are used to treat type II diabetes and they have beneficial effects on inflammation [157, 158]. Hence, the efficacy of the Rosiglitazone in ameliorating peritoneal membrane damage was tested in a mouse PD model. Rosiglitazone reduced peritoneal AGEs accumulation, preserved the mesothelial cell monolayer, reduced fibrosis and angiogenesis, and improved peritoneal ultrafiltration. This was associated with increased peritoneal concentration of the anti-inflammatory cytokine interleukin-10 (IL-10) and with a higher percentage of CD4/CD25/FoxP3 regulatory T cells (Tregs) [108]. These animal experiments provide proof-of-concept evidence for the feasibility and potential efficacy of targeting the inflammation in order to preserve the peritoneal membrane. The clinical use of some of the specific compounds tested so far in animals may encounter several hurdles. Thus, side effects associated with thiazolidinediones including edema, weight gain, bone fracture risk, heart failure, and an adverse lipid profile, have led to the withdrawal from the European market of Rosiglitazone [26, 108]. Prolonged use of COX-2 inhibitors may exert vasoconstrictor and thrombogenic effects, especially worrisome in renal patients, who have a high cardiovascular risk [26]. Immunomodulatory drugs may have an impact on the risk or severity of peritonitis. Further studies are needed in this regard since none of the *in vivo* PD studies addressed infectious complications. Still, independently of any specific drug considerations, preclinical studies support the feasibility of modulating inflammation pharmacologically to improve the response to bioincompatible PD fluids [26, 108, 148].

Tamoxifen is a synthetic modulator of the estrogen receptor that has been used successfully to treat retroperitoneal fibrosis and EPS associated with PD [52, 111]. Thus, the efficacy of Tamoxifen to preserve the peritoneal structure and function was tested in the mouse PD model. Oral administration of Tamoxifen significantly reduced peritoneal thickness, angiogenesis, invasion of the compact zone by mesenchymal MCs, and improved peritoneal function. Tamoxifen also reduced the effluent levels of VEGF and leptin [111]. In contrast to Celecoxib and Rosiglitazone that did not exert any effect on the MMT process *in vitro* [108, 148], Tamoxifen blocked the MMT induced by TGF-*β*1, as it preserved the expression of E-cadherin and reduced the expression of mesenchymal-associated molecules [111]. Tamoxifen also inhibited the invasion capacity of mesenchymal-like MCs by a mechanism implicating the inhibition of matrix metalloproteinase-2 (MMP-2) synthesis [111]. These results demonstrate that Tamoxifen is a therapeutic option to treat peritoneal fibrosis and that its protective effect is mediated via modulation of the MMT process.

Other examples of drugs that can be administrated either locally or by oral route are certain inhibitors of the renin-angiotensin-aldosterone system (RAAS) including Aliskiren, Valsartan, Enalapril, and Lisinopril (Figure 6). Components of the RAAS are constitutively expressed within peritoneal MCs and are upregulated in the presence of acute inflammation and chronic exposure to peritoneal dialysate. Furthermore, activation of the RAAS contributes to MMT, resulting in progressive fibrosis and angiogenesis of the peritoneal membrane [159]. Administration of the RAAS inhibitors by different routes reduced peritoneal thickening and improved peritoneal function in PDF exposure models in rats [159–162].

Activators of vitamin D receptor (VDR) are used to treat secondary hyperparathyroidism in PD patients. VDR activation modulates inflammation, fibrosis, and immune responses, modifying the Th1/Th2 pattern, inducing Tregs, and decreasing NF-*κ*B [163]. It also exerts antiproliferative actions, increases antifibrotic factors such as BMP-7, and decreases renal fibrosis [163]. However, the potential benefit of VDR activators for the peritoneum has not been studied so far.

The molecular characterization of the TGF-*β*1-mediated signaling and other pathways involved in the regulation of MMT provide a wide range of possible molecular targets such as ERKs-1/2, JNK, TAK 1, NF-*κ*B, and Notch 1, many of which still require to be tested in animal PD models [105, 112, 120] (Figure 6). In this regard, it has been shown that TGF-*β*1 induced Notch signaling in rat peritoneal MCs. The gamma-secretase inhibitor “DAPT” significantly inhibited *in vitro* the TGF-*β*1-induced expression of the mesenchymal markers *α*-SMA, collagen I, and VEGF. Furthermore, it has been demonstrated that intraperitoneal injection of DAPT significantly attenuated peritoneal fibrosis, decreased mass transfer of glucose, and increased ultrafiltration rate in a rat PD model. Thus, the gamma-secretase inhibitor that interferes with Notch signaling prevents biochemical, histological, and functional consequences of peritoneal fibrosis through inhibiting MMT [113].

Finally, an alternative therapeutic approach that still needs to be tested *in vivo* consists of the blocking of the invasive capacity of mesenchymal MCs to avoid their accumulation in the submesothelial compact zone (Figure 6). MMT is accompanied by upregulated expression of matrix metalloproteinases such as MMP-2 and MMP-9, which would degrade the basal membrane and the connective tissue allowing the submesothelial invasion by the mesenchymal-like MCs. It could be expected that MMPs inhibitors, or drugs that inhibit the synthesis of MMPs (e.g., Tamoxifen), may prevent the accumulation MCs-derived myofibroblasts in the submesothelial compartment. Recently, it has been demonstrated that invasion capacity of MCs that have undergone a MMT is governed, at least partially, by the VEGF/VEGF receptors/coreceptors axis [62]. It was shown that blocking antibodies directed against VEGF or the coreceptor neuropilin-1 efficiently interfered the invasion of MCs *in vitro* [62]. It would be interesting to test *in vivo* whether the prevention of the accumulation of MCs-derived myofibroblasts would, in turn, diminish the structural alteration of the peritoneal membrane.

## 8. Conclusions 

During the last years several studies using *ex vivo* cultures of effluent-derived MCs, in conjunction with immunohistochemical analysis of peritoneal biopsies, have allowed the identification of the MMT as a key process in peritoneal membrane failure. In fact, it could be demonstrated that effluent-derived MCs reflect the functional status of the peritoneal tissue of PD patients. It can be expected that different omics approaches applied to the MMT process will provide new biomarkers, with diagnostic and/or prognostic value, for the progressive peritoneal deterioration, and for the identification of master molecules governing the mesenchymal conversion of MCs.

Pharmacological interventions targeting MMT or MMT-promoting stimuli operating *in vivo* (e.g., inflammation) represent interesting approaches to limit peritoneal damage during PD. The feasibility of two pharmacological intervention approaches has been tested in experimental animal models of PD. One was the addition of pharmacological agents to the PD fluids. This approach has been useful for proof-of-concept studies. However, incorporation of new components to the PD solutions requires major changes from a regulatory point of view and will increase the cost of PD. Self-administration of a therapeutic agent into the solution by the patient will also increase the cost of PD and has a potential risk of contamination. The other approach, the use of oral agents, is technically easier. The general response to tissue injury involves inflammation to eliminate the insult as well as damaged tissue in order to restore its architecture and functionality. Sustained inflammation promotes fibrosis and angiogenesis, processes associated with the ultrafiltration failure that causes PD technique dropout. PD patients present a chronic inflammatory state and may suffer acute inflammatory processes induced by infection or “haemoperitoneum.” A better understanding of the role and regulation of inflammation in PD-related peritoneal damage is essential to design novel therapeutic strategies to protect the peritoneal membrane.

Careful benefit/risk studies are required. Ideally, we should better understand the potential benefits for the peritoneum of drugs that may serve multiple purposes for PD patients. Since a key market for these approaches is the low-income countries that cannot afford the newer, more biocompatible PD fluids, cost will be an issue and generic drugs are preferable over new compounds. In one scenario, patients may use the drug for as long as they are on PD. In other scenarios, the drugs would be required during especially vulnerable periods, as the peritonitis episodes or when hyperosmotic fluids are needed.
